# Epigenetic age predictions based on buccal swabs are more precise in combination with cell type-specific DNA methylation signatures

**DOI:** 10.18632/aging.100972

**Published:** 2016-05-31

**Authors:** Monika Eipel, Felix Mayer, Tanja Arent, Marcelo R. P. Ferreira, Carina Birkhofer, Uwe Gerstenmaier, Ivan G. Costa, Stefanie Ritz-Timme, Wolfgang Wagner

**Affiliations:** ^1^ Helmholtz-Institute for Biomedical Engineering, Stem Cell Biology and Cellular Engineering, RWTH Aachen University, Aachen, Germany; ^2^ Institute for Biomedical Engineering - Cell Biology, University Hospital of RWTH Aachen, Aachen, Germany; ^3^ Institute for Legal Medicine, Heinrich Heine University, Düsseldorf, Germany; ^4^ Department of Statistics, Centre for Natural and Exact Sciences, Federal University of Paraiba, 58051-900, João Pessoa, Brazil; ^5^ IZKF Computational Biology Research Group, University Hospital of RWTH Aachen, Aachen, Germany; ^6^ Varionostic GmbH, 89081 Ulm, Germany

**Keywords:** aging, predictor, epigenetic, methylation, swab, cell composition, epithelial cells

## Abstract

Aging is reflected by highly reproducible DNA methylation (DNAm) changes that open new perspectives for estimation of chronological age in legal medicine. DNA can be harvested non-invasively from cells at the inside of a person's cheek using buccal swabs – but these specimens resemble heterogeneous mixtures of buccal epithelial cells and leukocytes with different epigenetic makeup. In this study, we have trained an age predictor based on three age-associated CpG sites (associated with the genes *PDE4C*, *ASPA*, and *ITGA2B*) for swab samples to reach a mean absolute deviation (MAD) between predicted and chronological age of 4.3 years in a training set and of 7.03 years in a validation set. Subsequently, the composition of buccal epithelial cells versus leukocytes was estimated by two additional CpGs (associated with the genes *CD6* and *SERPINB5*). Results of this “Buccal-Cell-Signature” correlated with cell counts in cytological stains (R^2^ = 0.94). Combination of cell type-specific and age-associated CpGs into one multivariate model enabled age predictions with MADs of 5.09 years and 5.12 years in two independent validation sets. Our results demonstrate that the cellular composition in buccal swab samples can be determined by DNAm at two cell type-specific CpGs to improve epigenetic age predictions.

## INTRODUCTION

Estimation of chronological age of persons with (allegedly) unknown age is highly relevant in legal medicine – today more than ever. For example, such estimations are decisive for the legal status of young refugees in asylum procedures and for the degree of penalty for young offenders. During childhood and adolescence, sufficiently precise age estimates can be achieved by the assessment of skeletal and dental development with radiologic examinations - those examinations, however, have irradiation side effects and are not permissible in all legal systems. Other precise methods that can be used, such as the biochemical analysis of the aspartic acid racemization of tooth dentin, are not generally applicable for living individuals [1]. Molecular parameters, such as telomere shortening [2], T-cell DNA-rearrangements [3], or mitochondrial deletions provide only relatively low accuracy. In this regard, the recently developed approaches of using age-associated epigenetic modifications for age estimation appear to be promising [4-6].

DNA methylation (DNAm) is so far the best understood epigenetic modification [7]. It has been suggested that almost one third of CpG dinucleotides reveal age-associated modifications on the DNAm level [8]. Hypermethylation or hypomethylation are almost linearly acquired with age at some CpGs – at least in adult donors - and can therefore be used for age predictions. For example Hannum et al. used global DNAm profiles of blood samples of a large cohort to derive a multivariate linear model based on 71 CpGs [5]. This approach facilitated age predictions with a mean error of 4.9 years in independent blood samples, but it had a clear offset in other tissues. On the other hand, it has been demonstrated that several age-related CpGs reflect similar changes across different cell types and tissues [5, 9, 10]. Cell type-specific effects can partly be compensated by a higher number of age-associated CpGs: a multi-tissue predictor based on 353 age-associated CpG sites was developed by Horvath [6] that enables age-estimations for a wide range of cell types. However, simultaneous analysis of DNAm in hundreds of CpGs is only feasible with profiling technologies, such as Illumina Bead Chip microarrays or deep sequencing, making it difficult to implement this approach for efficient high-throughput analysis in daily routine of legal medicine. We have recently developed an epigenetic age predictor based on DNAm levels at just three age-associated CpGs located in the genes integrin alpha 2b (*ITGA2B*), aspartoacylase (*ASPA*), and phosphodiesterase 4C (*PDE4C*) [4]. DNAm levels at these CpGs can be analyzed site-specifically with cost-effective, fast and reliable pyrosequencing assays to provide age predictions with a mean absolute deviation (MAD) from chronological age of less than 5 years in blood samples – so far, application of the three-CpG signature on other tissues has not been described.

Buccal swabs are widely used specimens in legal medicine due to their non-invasive and convenient harvesting procedure. In principle, Bocklandt and coworkers have demonstrated that saliva samples can be used for epigenetic age predictions [11]. The authors used three CpGs associated with the genes EDAR-associated death domain (*EDARADD*), neuronal pentraxin II (*NPTX2*), and target of myb1 like 1 membrane trafficking protein (*TOM1L1*) to predict age with a MAD between predicted and chronological age of 5.2 years, but the precision was not validated on an independent set of samples. Saliva as well as mouth swabs are very heterogeneous in their composition of buccal epithelial cells and leukocytes [12] and it can be anticipated that the epigenetic makeup as well as age-associated changes differ significantly between these two cell types. In this study, we therefore followed the hypothesis that the precision of epigenetic age predictions in buccal swabs can be improved by taking the cellular composition of buccal epithelial cells *versus* leukocytes into account.

## RESULTS

### Retraining epigenetic age predictors for buccal swabs

**Figure 1:**
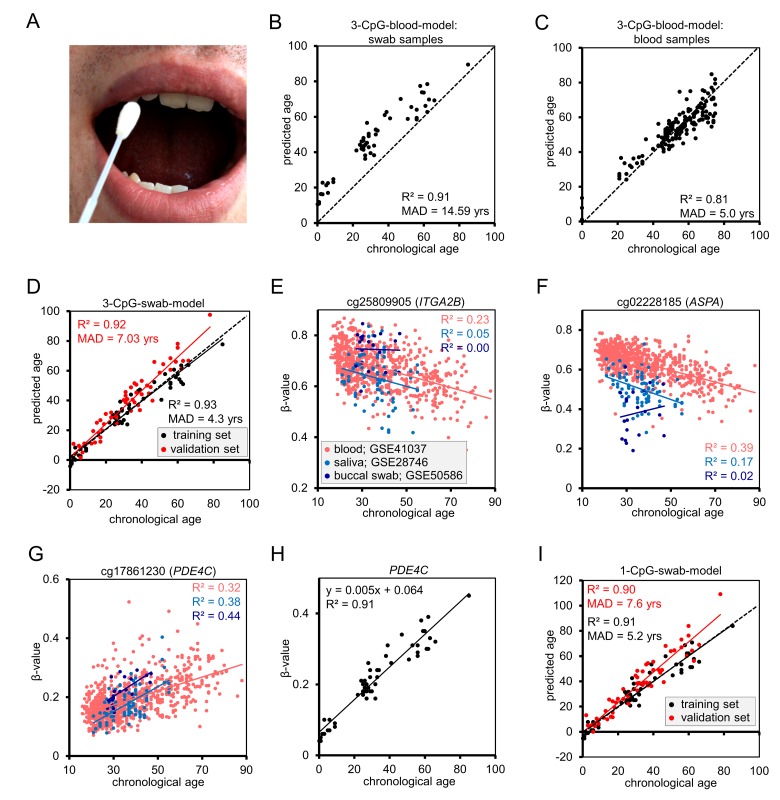
Epigenetic aging model for blood needs to be adjusted for buccal swabs **(A)** Illustration of sample collection with a buccal swab. **(B)** Epigenetic age predictions of 55 mouth swab samples using an age predictor that was trained on blood samples as described before [4]. **(C)** For comparison, we demonstrate the predictions for 151 whole blood samples of our previous work [4]. **(D)** The multivariate model for age predictions was then retrained on pyrosequencing results of 55 mouth swab samples and validated on 55 independent additional samples that were analyzed in a different lab. **(E-G)** Correlation of β-values of age-associated CpG sites with chronological age. To this end, we used publically available datasets of blood (GSE41037), saliva (GSE28746), and mouth swabs (GSE50586). The CpG site cg17861230 corresponds to the neighboring CpG site in PDE4C that was used in the pyrosequencing models (because, the latter is not represented by Illumina Bead Chips). **(H)** β-values of the CpG site in the PDE4C gene in swab samples were determined by pyrosequencing and correlated with chronological age. **(I)** Age predictions based on DNAm levels at the CpG site in *PDE4C*. The linear regression model is depicted in (H). MAD = mean absolute deviation.

Buccal swab samples were taken from 55 healthy donors (age range of 1 to 85 years; Figure 1A) and DNAm levels were analyzed by pyrosequencing at the three relevant age-associated CpGs of our blood-based age predictor [4], subsequently referred to as “3-CpG-blood-model”. The correlation of predicted and chronological age was R^2^ = 0.91 (Pearson correlation), and this was even slightly higher than previously observed in 151 blood samples (R^2^ = 0.81; Figure 1B-C) [4]. However, there was a clear offset in age predictions of buccal swabs: in average buccal swab samples were overestimated by 14.6 years. Therefore, we retrained the multivariate model on the pyro-sequencing results of the 55 buccal swab samples as follows: Predicted age (years) = 32.70 – 8.42 (β-value of cg02228185) – 47.38 (β-value of cg25809905) + 183.25 (β-value of CpG upstream of cg17861230). The MAD was only 4.3 years in the training set (R^2^ = 0.93; Figure 1D) and this model is subsequently referred to as “3-CpG-swab-model”. We have validated this model on an independent validation set of 55 swab samples that were taken and analyzed in other labs and in different towns – here, the MAD was 7.03 years (R^2^ = 0.92; Figure 1D). Notably, epigenetic age of the validation set was systematically over-estimated, which might be attributed to differences in the harvesting procedure or slight differences in pyrosequencing measurements in the different labs.

To gain better insight into tissue-specificity of individual CpGs, we compared publically available DNAm profiles of blood, saliva, and mouth swab samples (GSE41037 [6], GSE28746 [11] and GSE50586 [13], respectively). In buccal swabs and saliva samples we hardly observed linear correlation between β-values and chronological age at the CpGs in *ASPA* (cg02228185) and *ITGA2B* (cg25809905) – they may therefore not be ideal candidates for age-associated biomarkers in buccal swabs. In contrast, the CpG site in *PDE4C* (cg17861230) demonstrated even higher correlation with chronological age in saliva and buccal swabs as compared to blood (Figure 1 E-G). This was also confirmed in our pyrosequencing analysis (R^2^ = 0.91; Supplemental figure S1). Therefore, we reasoned that the CpG site in *PDE4C* might be sufficient for reliable age predictions: linear regression of DNAm levels in *PDE4C* was used as a more convenient “1-CpG-swab-model” with a MAD of 5.2 years in the training set (R = 0.91) and 7.6 years in the validation set (R = 0.90; Figure 1H,I).

### Analysis of the composition of buccal epithelial cells versus leukocytes

**Figure 2:**
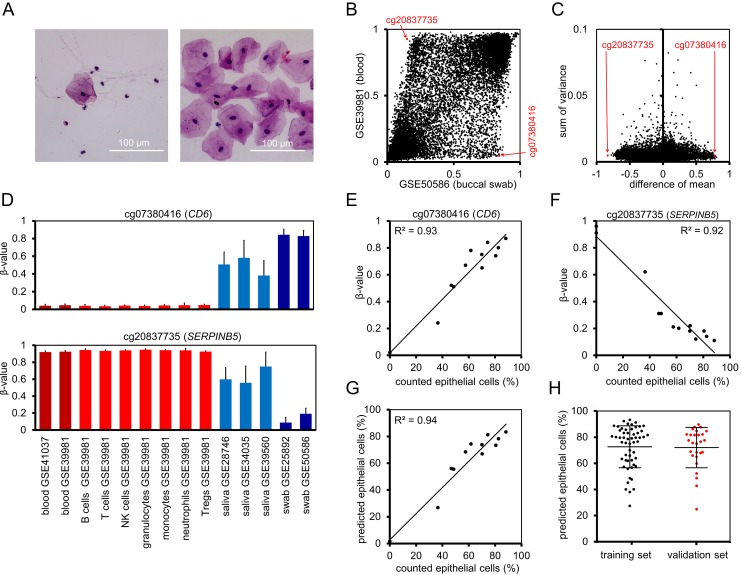
Prediction of the cellular composition in mouth swab samples **(A)** Representative mouth swab smears with different proportions of leukocytes and epithelial cells. Smears of freshly harvested cells were stained with haematoxylin and eosin. **(B)** Mean β-values of CpGs on Illumina 27k Bead Chip in datasets of buccal swabs (GSE50586) and blood (GSE39981). Red arrows indicate CpG sites selected for the “Buccal-Cell-Signature”. **(C)** As additional criterion for suitable cell type-specific CpGs we used the sum of variances in both datasets. **(D)** Mean β-values at cg07380416 (CD6) and cg20837735 (SERPINB5) were compared in whole blood (GSE41037, GSE39981), hematopoietic subsets (GSE39981), saliva (GSE28746, GSE34035, GSE39560), and buccal swabs (GSE25892, GSE50586). Error bars represent standard deviation. **(E, F)** The percentage of buccal epithelial cells versus leukocytes was determined by cell counting in 11 stained mouth swab smears. DNAm levels at the two cell type-specific CpGs were determined by pyrosequencing and correlated with cell counts. **(G)** Linear regressions of both CpGs were combined into the Buccal-Cell-Signature. Predicted percentages of buccal epithelial cells correlated with cell counts. **(H)** Percentages of epithelial cells were subsequently estimated using the Buccal-Cell-Signature for 55 samples of the training set and 26 samples of the validation set. Error bars represent standard deviation.

Mouth swab samples comprise particularly buccal epithelial cells and leukocytes. The proportions of cell types may vary, e.g. due to harvesting procedures [12]. We determined the fractions of leukocytes and buccal epithelial cells in 11 mouth swab samples by cell counting in haematoxylin/eosin stained smears (Figure 2A): the proportion of leukocytes varied between 12% - 63% (mean of 35%). This is in line with a previous study based on short tandem repeats after allogeneic hematopoietic stem cell transplantation that described percentages of leukocytes between 5% - 60% in buccal swabs and 16% - 95% in mouthwash samples [12].

We reasoned that epigenetic characteristics of buccal epithelial cells and leukocytes might be utilized to determine the cellular composition in buccal swabs. To identify suitable CpGs we used DNAm datasets of swabs (GSE50586) [13] and whole blood samples (GSE39981) [14] to filter with the following criteria: i) high difference in mean DNAm levels in swabs and blood (Figure 2B), ii) low variance in DNAm levels within each of these datasets (Figure 2C), and iii) no correlation with chronological age in blood samples of 656 donors, aged 19 to 101 (GSE40279), [5]; Pearson correlation < 0.05; Supplemental Figure S2). Furthermore, we validated our selection on two independent datasets from buccal swabs GSE25892 [15] and blood GSE41037 [16] (Supplemental Figure S3). Based on these parameters, we identified a CpG site associated with the gene for T-cell differentiation antigen *CD6* (cg07380416) and a CpG site in the gene for serpin peptidase inhibitor clade B member 5 (*SERPINB5*; cg20837735) as best suited candidates. The distribution of β-values was further analyzed in DNAm profiles of various hematopoietic cell types: cg07380416 was consistently hypomethylated, whereas cg20837735 was hypermethylated across the different types of blood cells (Figure 2D). Mean DNAm levels in saliva, which generally comprise higher numbers of leukocytes than swabs, were between those of blood and swabs. Furthermore, neighboring CpGs of cg07380416 and cg20837735 demonstrated similar differences between the cell types (Supplemental Figure S4), indicating that the two genomic regions might be suitable to reliably estimate the cellular composition of buccal epithelial cells and leukocytes.

Subsequently, we designed pyrosequencing assays for the two relevant CpGs (Supplemental Figure S5) and tested the 11 buccal swab samples that were analyzed by cytological stains as well as two additional blood samples: in fact, the β-values in cg07380416 (*CD6*) and cg20837735 (*SERPINB5*) correlated with the proportion of counted epithelial cells (R^2^ = 0.93 and R^2^ = 0.92, respectively; Figure 2E,F), indicating that both CpGs adequately reflect the cellular composition. The two CpGs were then combined into a model that is subsequently referred to as “Buccal-Cell-Signature”: Percentage of buccal epithelial cells (ϐ) = (99.8 (β-value of cg07380416) + 1.92) / 2 + (−98.12 (β-value of cg20837735) + 88.54) / 2.

The predicted fractions of epithelial cells correlated with the counted cell fractions (R^2^ = 0.94; Figure 2G). We then utilized the Buccal-Cell-Signature for analysis of buccal swab samples of 55 samples of the training set and 26 samples of the validation set. The predicted fraction of buccal cells ranged between 24% and 91% (mean 71%). This analysis was performed in the same lab for all samples to exclude technical variation in pyrosequencing analysis. There was no significant difference between training and validation set (Figure 2H), indicating that the above mentioned moderate overestimation of age in the validation set is not due to different harvesting procedures.

### The impact of smoking, ethnicity and gender

**Figure 3:**
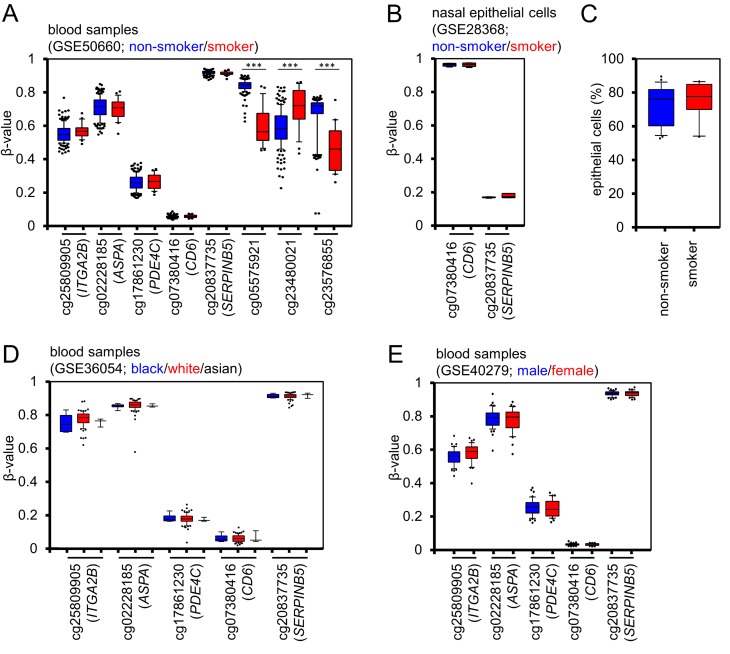
Smoking, ethnicity and gender do not impact on DNAm at selected CpG sites **(A**) DNAm levels at the three age-associated CpG sites (*ITGA2B*, *ASPA*, and *PDE4C*), and the two cell type associated CpGs (CD6 and SERPINB5) did not differ in blood samples of current smokers (red) and never-smokers (blue; GSE50660). In contrast, such differences were validated in three CpG sites, which have previously been described as smoking-associated. **(B)** DNAm profiles of pure nasal epithelial cells of smokers (red) and non-smokers (blue) did not demonstrate differences in the two cell type associated CpGs (GSE28368). **(C)** Pyrosequencing analysis of the Buccal-Cell-Signature in 36 samples with known smoking status did not reveal differences in the cellular composition of buccal swabs. **(D)** DNAm profiles of children (1 to 17 years) did not reveal significant differences between different ethnic groups (GSE36054; blue: black donor; red: white donor; black lines: Asian donor). **(E)** None of the five CpGs revealed gender-associated differences (GSE40279; blood samples of 40 to 50 year old donors; blue: female; red: male). * P < 0.05; *** P < 0.0005; Whiskers indicate 10% and 90% percentiles, respectively.

Confounding factors - such as smoking, ethnicity, and gender – can impact on DNAm levels at specific sites in the genome [17-20]. Therefore, we tested if our age-associated or cell type-associated CpG sites are systematically influenced by these parameters. The β-values did not differ in blood samples of 22 smokers and 179 non-smokers (GSE50660, similar age distribution [21]). In contrast, we could recapitulate significant changes at previously described smoking-associated CpG sites (Figure 3A) [20-22]. Furthermore, there was no effect of smoking on the CpGs of the Buccal-Cell-Signature in DNAm profiles of nasal epithelial cells of 6 smokers and 6 non-smokers – but they clearly reflected the epithelial cell type (GSE28368, data on chronological age was not available [23]; Figure 3B). Subsequently, we compared the results of our Buccal-Cell-Signature in 26 known non-smokers and 10 smokers and found no evidence that smoking affected the composition of buccal epithelial cells *versus* leukocytes in buccal swabs (Figure 3C). To estimate if DNAm levels at our five CpGs differ between ethnical groups we analyzed DNAm profiles of 8 white, 74 black and 3 Asian children (GSE36054, [24]) and there were no significant differences (Figure 3D). In analogy, we compared DNAm levels in male and female samples and found no gender-associated variation (GSE40279, 40 – 50 year old donors, [5]) (Figure 3E). These results indicate that smoking, ethnicity, and gender hardly affect our predictions of epigenetic age or of the cellular composition.

### Combination of cell type-specific and age-associated CpGs into one model

**Figure 4:**
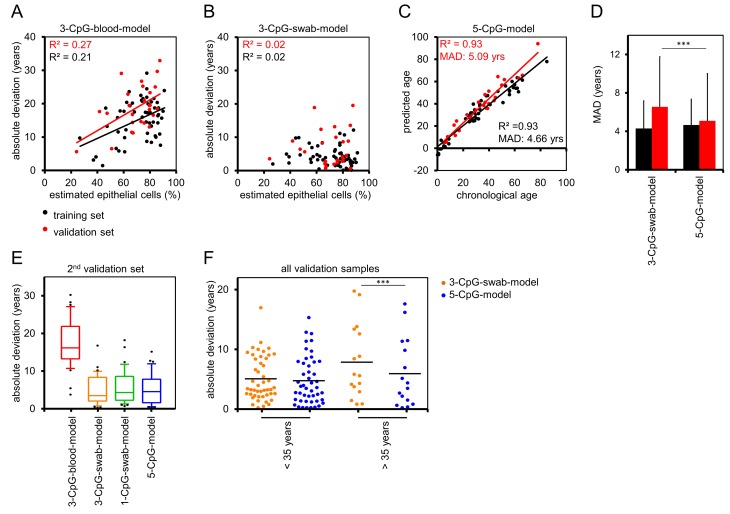
Buccal-Cell-Signature improves epigenetic age prediction **(A)** The differences of chronological age and predictions by the 3-CpG-blood-model were compared to the predicted percentage of buccal epithelial cells (according to the Buccal-Cell-Signature). Deviations were higher in samples with more buccal epithelial cells. **(B)** In analogy, we compared age predictions by the 3-CpG-swab-model to the estimated percentage of buccal epithelial cells and here the impact of the cellular composition was less clear. **(C)** Combination of age-associated CpGs and Buccal-Cell-Signature in a multivariate regression model of five CpGs (5-CpG-model) facilitated age predictions in the training and validation set. **(D)** Mean absolute deviations of predicted and chronological age were significantly smaller in the validation set when using the 5-CpG-model as compared to the 3-CpG-swab-model. **(E)** The models for age-prediction were subsequently validated in a second, independent dataset of 37 samples (18 to 35 years). **(F)** Samples of the validation group were stratified by an age of 35 years. Comparison of the 3-CpG-swab-model and the 5-CpG-model revealed that the additional analysis of the Buccal-Cell-Signature was particularly relevant for samples of older donors (*** P < 0.0005).

To test if the precision of epigenetic age predictions is affected by the cellular composition in buccal swabs, we compared the estimated fraction of buccal epithelial cells *versus* the MAD of predicted and chronological age. In fact, the offset of age predictions in the training and validation datasets by the 3-CpG-blood-model was higher in samples with a higher fraction of epithelial cells (Figure 4A). These cell type-specific differences were less pronounced when using the 3-CpG-swab-model (Figure 4B).

Subsequently, we followed the hypothesis that age predictions can be improved by taking the cellular composition into account. We combined the age-associated CpGs and the Buccal-Cell-Signature into one linear “5-CpG-model”. This adjusted model gave age predictions with a MAD from chronological age of 4.66 years (R^2^ = 0.93) in 55 samples from the training set and of 5.09 years (R^2^ = 0.93; Figure 4C) in the 26 samples of the validation set. These results were more precise in the validation set than predictions by the 3-CpG-swab-model (P = 2.2×10^−4^, Figure 4D). We then asked whether age prediction accuracy is comparable in young and elderly donors. Therefore, we divided all samples into two groups stratified by an age of 30 years. Overall, the precision of epigenetic age predictions was even higher in younger donors (Supplemental Figure S6).

To further evaluate the precision of our models we analyzed buccal swabs of an additional independent set of 37 donors between 18 and 35 years. As described above, the 3-CpG-blood-model would highly over-estimate donor age (MAD = 17.3 years), whereas the epigenetic age-predictions of the 3-CpG-swab-model (MAD = 4.84 years), the 1-CpG-swab-model (MAD = 5.6 years), and the 5-CpG-model (MAD = 5.12 years) further substantiated the relatively high precision of our signatures (Figure 4E). It was however unexpected that the 5-CpG-swab-model did not outperform the 3-CpG-swab-model in these samples.

This can be attributed to the fact, that DNAm levels of *PDE4C* are anyway quite similar in blood and buccal swabs at this age range (Figure 1G). When we stratified samples of the validation groups according to an age of 35 years, it became evident that effects of cell type adjustment by the Buccal-Cell-Signature are particularly important at higher ages (P = 0.0003; Figure 4F).

## DISCUSSION

Age-associated DNAm changes are acquired in a similar fashion in different cell types and tissues [6, 10, 25], but the difference in the epigenetic makeup of different cell types undoubtedly affects epigenetic age predictions. It has been demonstrated that precision of epigenetic age predictions of blood samples can be improved by taking blood counts into consideration [26]. Accordingly, it can easily be assumed that buccal epithelial cells and blood cells – which greatly differ in morphology, function, and derivation – have very pronounced differences in their DNAm patterns. Our data indicate that some age-associated CpGs are less affected by the cellular composition than others. To better identify suitable CpGs it would be valuable to utilize DNAm profiles of purified buccal epithelial cells of young and elderly donors – however, such datasets are so far not available making it difficult to estimate cell type-specific age-associated modifications.

In this study, we describe a new method to determine the composition of buccal epithelial cells *versus* leukocytes by measuring DNAm levels at two CpG sites. Both CpGs revealed high correlation with the leukocyte counts (R^2^ = 0.93 and 0.92). Combination of one methylated and one non-methylated CpG into one model enables internal quality control and more robust measurements. We have recently described a similar regimen of two CpG-signatures to categorize pluripotent and non-pluripotent cells [27], to distinguish between mesenchymal stromal cells (MSCs) and fibroblasts [28], and to determine if MSCs were isolated from either adipose tissue or bone marrow [28]. The Buccal-Cell-Signature could also be used to estimate the cellular composition in saliva samples, which usually comprise even higher fractions of leukocytes. Furthermore, the method might be useful for analysis of unknown body fluids, or to gain insight into the harvesting procedure of buccal swabs.

Particularly for application in legal medicine it is important to better understand how epigenetic age predictions are affected by additional parameters such as local infections, diseases affecting growth and development, or obesity [29-31]. Our exploratory analysis indicated that smoking, ethical background, and gender hardly affect DNAm levels at the five relevant CpG sites. However, further analysis in larger cohorts and for additional parameters will be necessary to gain better insight on how these or other clinical parameters impact on epigenetic age predictions.

Many groups have described epigenetic age predictors that are based on few or even individual CpGs [32-34] and some of these have been used for saliva samples [11] – however, to our knowledge they have so far not been applied for buccal swab samples. The precision of epigenetic signatures can generally be increased by implementing a larger number of CpGs [6, 35]. As mentioned above, the predictor of Horvath was trained on 353 CpGs of Illumina Bead Chip data to work robustly on samples of multiple tissues – thus, larger models that utilize many more CpGs may not require specific adaptation to the cellular composition. On the other hand, even such large aging signatures could be combined with cell type-specific signatures. The cell type-specific information may provide quality control and help to further refine the precision of epigenetic age-predictions – by using a similar mathematical regimen as exemplified in our study.

In contrast to analysis of global DNAm profiles, our signature can be addressed by site-specific assays, such as pyrosequencing or MassARRAY, which facilitate more quantitative measurements [35]. These assays are cost-effective, enable analysis within days and do not require complicated bioinformatics. For validation, we have used an independent set of samples that has been harvested at a different University, at a different time, and analyzed in a different lab – in this regard we have used the most stringent validation possible and the results support the notion of high reproducibility. However, even after correction for the cellular composition the age predictions in the validation cohort were in tendency over-estimated. Thus, there may be a small systematic bias by pyrosequencing analysis in different labs that should be taken into account. It has also been suggested that in childhood most age-associated changes should rather be modeled as a function of logarithmic age [24]. Notably, our data demonstrate that the precision of our aging signatures was also relatively high in children and young adults. This is important, as it resembles exactly the age-range that is particularly relevant in legal medicine. On the other hand, the advantage of the Epithelial-Cell-Signature became evident in samples of elderly donors. In this study, we estimated the parameters for age-associated changes in the subfraction of buccal epithelial cells by subtraction of predictions for the blood subfraction in 55 samples – it is therefore expected that the 5 CpG model can be further improved on either a much larger number of samples, or by measurements of age-associated DNAm changes in purified buccal epithelial cells.

There is evidence that the epigenetic age rather reflects biological age than chronological age: the difference between predicted and chronological age is associated with cancer onset and overall survival [36-38]. Epigenetic age predictions may therefore support identification of relevant parameters for the aging process and thereupon adaptation of habits that assist healthy aging. Notably, the neighboring CpG site in the *PDE4C* gene was also found to be indicative for life expectancy in the Lothian Birth Cohort 1921 [35]. However, in legal medicine it is rather important to estimate chronological age. It is conceivable, that some age-associated CpGs are more biased by biological age than others – but this needs to be validated in the future [35].

In conclusion, buccal swabs resemble a suitable specimen for epigenetic age predictions – to either estimate chronological age in legal medicine or to gain additional insight into biological age. The composition of buccal epithelial cells and leukocytes can be estimated based on DNAm at one or two cell type-specific CpG sites. Such cell type-specific signatures can improve the precision of epigenetic age predictions and they might also improve other types of epigenetic diagnostics based on buccal swabs.

## METHODS

### Sample collection

All samples were taken after written consent and according to the guidelines of the local ethics committees. Blood samples were taken at the University Hospital in Aachen, Germany (ethics approval number EK 041/15). Buccal swab samples were collected at the University Hospital of RWTH Aachen (85 samples; EK 041/15) using FLOQSwabs (Copan Flock Technologies, Brescia, Italy) and by the Institute for Legal Medicine of the Heinrich Heine University in Düsseldorf, Germany (62 samples; study number #4939) using Mastaswabs (Mast Group ltd., Reinfeld, Germany). Samples were stored for up to 24h at room temperature and then at −20°C until they were further processed for DNA isolation (up to 2 weeks).

### DNA isolation and pyrosequencing

Genomic DNA was isolated with the NucleoSpin Tissue (Macherey und Nagel, Düren, Germany) and the QIAamp DNA Blood Mini Kit (Qiagen, Hilden, Germany) according to the manufacturer's instructions. Subsequently, 500 ng DNA were bisulfite-converted using the EZ DNA Methylation Kit (Zymo, Irvine, USA). Pyrosequencing of the three age-associated CpGs was performed as described in detail before [4]. Pyrosequencing of the age-associated CpGs of the training set were taken and processed at RWTH Aachen University; pyro-sequencing of age-associated CpGs of the first validation set (taken at Düsseldorf University) were processed by Cygenia GmbH (Aachen, Germany; www.cygenia.com); samples of the second validation set were taken and analyzed at both locations. The Buccal-Cell-Signature could not be applied to all samples of the first validation set as DNA was no more available. Further information on the pyrosequencing assays and primer information is provided in Supplemental Figure S6 and in Supplemental Table S1, respectively.

### DNA methylation datasets used in this study

To identify cell type-specific CpGs in blood, buccal swabs, and saliva samples, we utilized the following publically available DNAm datasets (all generated on the HumanMethylation27 and HumanMethylation450 BeadChip platforms): for blood GSE41037 (n = 720)[16], GSE40279 (n = 656) [5] and GSE39981 (n = 27, only whole blood) [14]; for saliva GSE28746 (n = 84) [11], GSE34035 (n = 197) [39], GSE39560 (n = 34) and for buccal swabs GSE25892 (n = 106; three DNAm profiles from this datasets were not considered as they resemble technical replica) and GSE50586 (n = 10; only healthy control samples) [13].

### Cytological analysis of cellular compositions in buccal swab samples

Smears of freshly taken swab samples were fixed with M-Fix^TM^ spray fixative (Merck, Darmstadt, Germany) according to the manufacturer's instructions. Cells were stained with Hematoxylin & Eosin (Merck, Darmstadt, Germany) or with Wright-Giemsa stain (Sigma-Aldrich, St.Louis, USA). Epithelial cells and leukocytes could easily be discerned by their morphology. For each sample we analyzed 50 randomly taken microscopic fields (corresponding to 328 ± 144 cells; cell counting was performed independent of pyrosequencing results).

### Derivation of epigenetic models

We used different linear models in this study that were all based on β-values determined by pyrosequencing for the following age-associated CpGs: (α) = cg02228185; (β) = cg25809905, and (γ) = a CpG site up-stream of cg17861230 which revealed better correlation with age [4]. In addition, we utilized two cell type-specific CpGs: (δ) = cg07380416 and (ε) = 20837735.

3-CpG-blood-model: This multivariate model has been described in detail in our previous work [4]. It was based on pyrosequencing results of 82 blood samples: Predicted age (in years) = 38.0 − 26.4 α − 23.7 β +164.7 γ.

3-CpG-swab-model: In analogy, we trained a similar multivariate model based on 55 swab samples of the training set: Predicted age (in years) = 32.69 − 8.42 α − 47.38 β + 183.25 γ.

1-CpG-swab-model: Alternatively, we used the linear regression line as 1-CpG model based on the CpG site associated with *PDE4C*: Predicted age (in years) = (γ − 0.0648) / 0.0046.

Buccal-Cell-Signature: We combined the linear regressions of the individual cell type-specific CpG sites into one model: Percentage of buccal epithelial cells (ϐ) = (99.8 δ + 1.9) / 2 + (−98.1 ε + 88.5) / 2.

5-CpG-swab-model: We assume that the model for prediction of age in buccal cells can be estimated by an additive model with predictions by the Buccal-Cell-Signature, i.e. predicted age blood * (1 − ϐ / 100) + Predicted age buccal epithelial * (ϐ / 100). By using ϐ estimated with the Buccal-Cell-Signature and predicted age blood corresponds to the 3-CpG-blood-model, we estimate parameters of age-associated linear models of buccal epithelial cells based on the 55 swab samples of the training set using R. This led to the following model: Predicted age (in years) = (1 − ϐ / 100) * (38.0 − 26.4 α − 23.7 β +164.7 γ) + (ϐ / 100) * (2.6 − 11.0 α − 15.6 β + 181.7 γ).

### Statistics

Error bars indicate standard deviations (SD). The paired two-sided Student's T-test was adopted to estimate the probability of differences in age prediction of different models, between smokers and non-smokers, and between male and female samples. Differences between ethnic groups were estmitated by an univariate ANOVA test. Probability value of P < 0.05 denotes statistical significance.

## SUPPLEMENTAL DATA


