# Supplementary material for: Epigenetic age predictions based on buccal swabs are more precise in combination with cell type-specific DNA methylation signatures
Source: Aging (Albany NY). 2016 May 31;8(5):1034–44. doi: 10.18632/aging.100972 (PMC4931852; doi:10.18632/aging.100972)
Supplement: Supplementary file 1 [file aging-08-1034-s001.pdf]

## SUPPLEMENTAL DATA

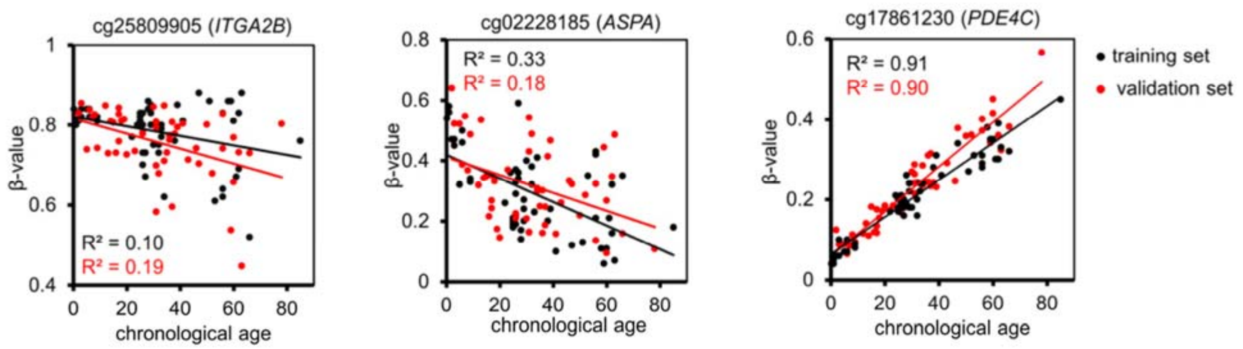


**Figure S1. Age-association of individual CpG sites in mouth swab samples.** Beta-values of the CpG sites in the genes ITGA2B, ASPA and PDE4C were determined by pyrosequencing and correlated with chronological age. Samples from the training and validation datasets are indicated in black and red, respectively.

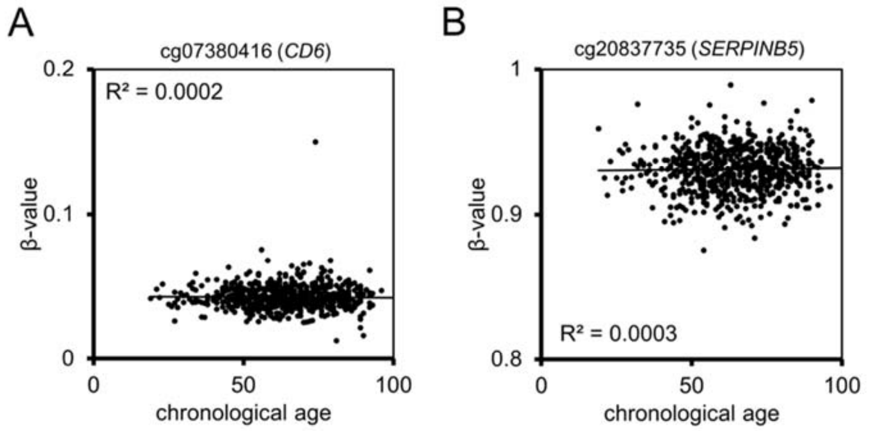


**Figure S2. Cell type-specific CpGs are not age-associated.** DNA-methylation levels at the CpG sites in **(A)** *CD6* and **(B)** *SERPINB5* were analyzed in blood samples of different chronological age (GSE40279). There was o clear association with chronological age, indicating that the selected.

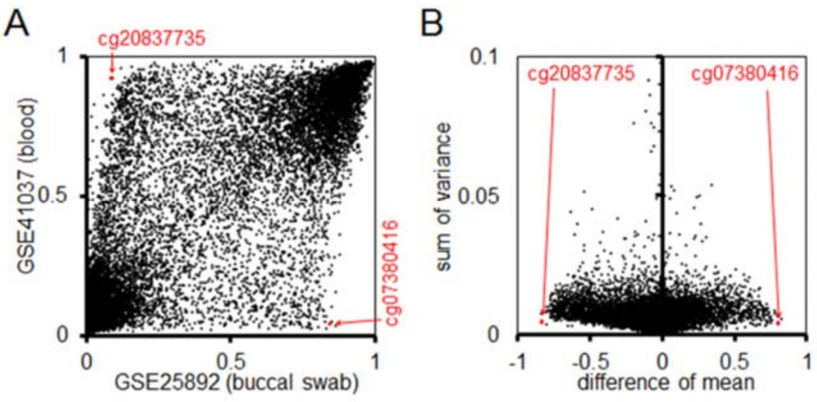


**Figure S3. Validation of cell type-specific CpGs using independent datasets. (A)** Mean DNAm levels in samples of buccal swabs (GSE25892) and blood (GSE41037; in analogy to Figure 2B). **(B)** The difference of mean s-values of individual CpG sites plotted *versus* the sum of variance (in analogy to Figure 2C). The highlighted two CpGs reveal also striking differences in these independent datasets.

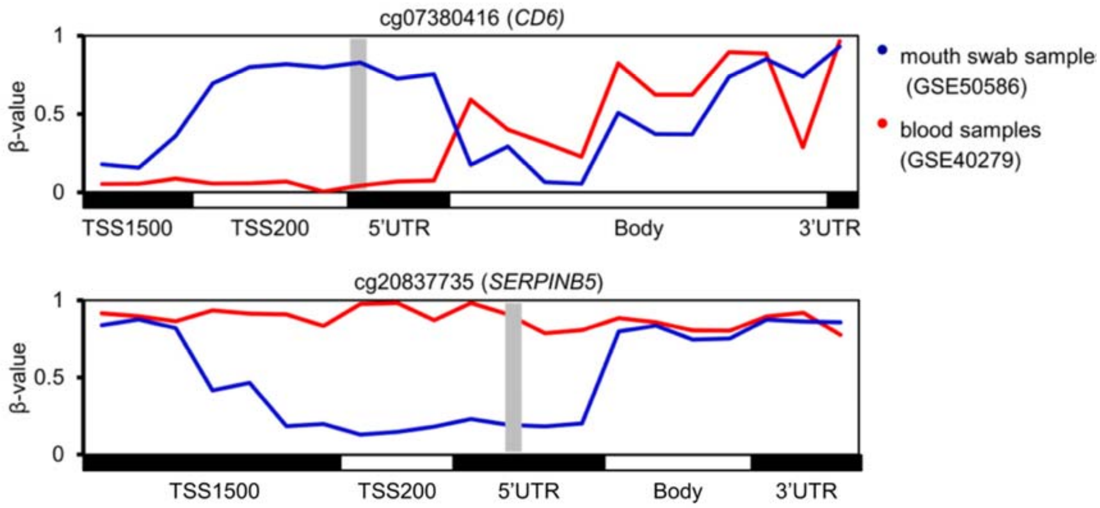


**Figure S4. DNAm patterns of CpGs in *CD6* and *SERPINB5*.** Mean β-values of all CpGs that are represented on the 450k BeadChip for the respective genes are depicted for blood (red lines; SE40279) and mouth swab samples (blue lines; GSE50586). Grey bars highlight the selected CpG sites. The neighboring CpGs also reveal cell-type specific differences. TSS1500: within 1500 bp of the transcription start site; TSS200: within 200 bp of the transcription start site; UTR: untranslated region.

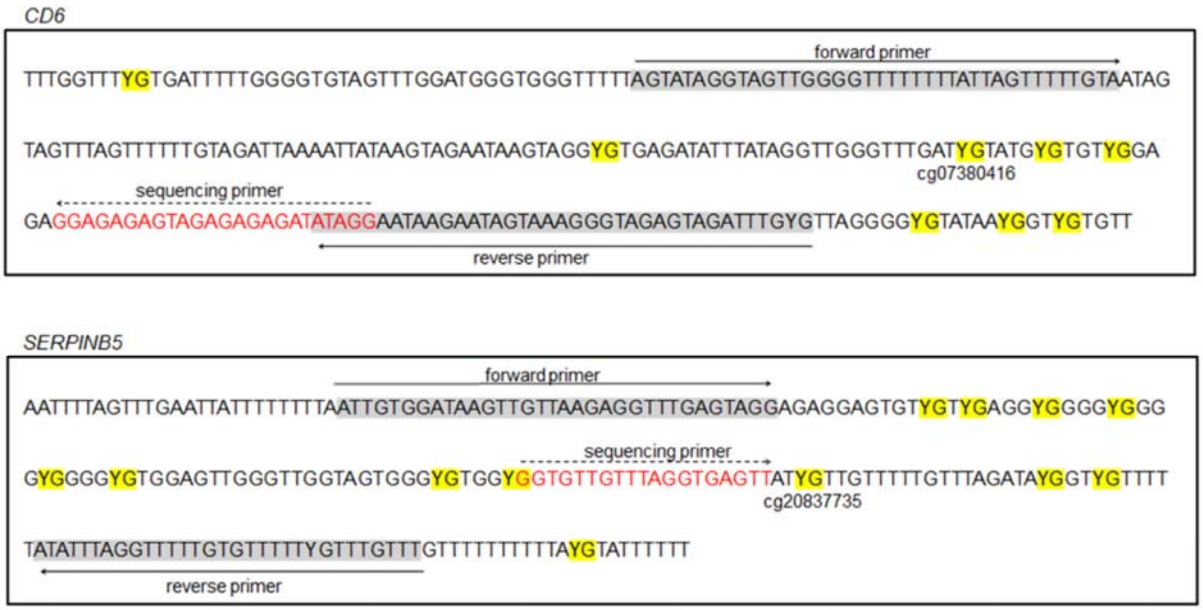


**Figure S5. Pyrosequencing assays for the Buccal-Cell-Signature.** Bisulfite converted sequence information for the CpG sites cg07380416 (*CD6*) and cg20837735 (*SERPINB5*). Arrows indicate sites for primer annealing and direction of elongation. Yellow indicates CpG sites after bisulfite conversion.

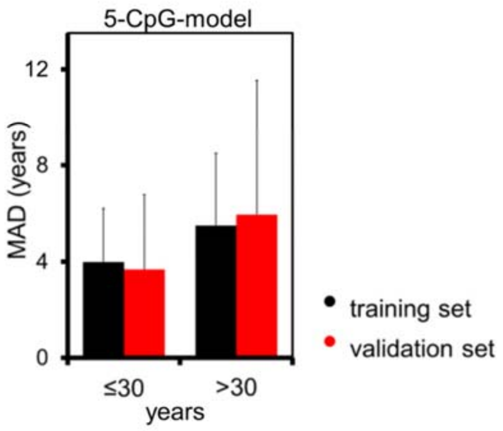


**Figure S6. Age prediction accuracy stratified by age.** To test if age predictions have similar accuracy in young donors as in older donors we divided all samples into two groups (stratified by an age of 30 years). Mean absolute deviations (MAD) for the training (black) and validation dataset (red) were in tendency more precise in younger donors. Error bars indicate standard deviations.

**Table S1. Primer sequences for pyrosequencing assays**

| CpG site | Location | Sequence |
|---|---|---|
| cg25809905 (*ITGA2B*) | Forward | Biotin-TAATTTTTTTTGGGTGATG |
| | Reverse | ACCAAAAATAAACAATATACTCAAT |
| | Sequencing | CAATATACTCAATACTATACCT |
| cg02228185 (*ASPA*) | Forward | Biotin-ATTATTTGGTGAAATGATT |
| | Reverse | CAACCCTATTCTCTAAATCTC |
| | Sequencing | CCCTATTCTCTAAATCTCA |
| cg17861230 (*PDE4C*) | Forward | AGGTTTGTAGTAGGTTGAG |
| | Reverse | Biotin-AACTCAAATCCCTCTC |
| | Sequencing | GTTATAGTATGATTAGAGTTT |
| cg07380416 (*CD6*) | Forward | Biotin-AGTATAGGTAGTTGGGGTTTTTTTTATTAGTTTTTGTA |
| | Reverse | CCAAATCTACTCTACCCTTTACTATTCTTATTCCTAT |
| | Sequencing | CCTATATCTCTCTCTACTCTCTCC |
| cg20837735 (*SERPINB5*) | Forward | ATTGTGGATAAGTTGTTAAGAGGTTTGAGTAGG |
| | Reverse | Biotin-AAACAAACAAACCAAAAACACAAAAACCTAAATAT |
| | Sequencing | GGTGTTGTTTAGGTGAGTT |
